# Identifying the Key Genes in Mouse Liver Regeneration After Partial Hepatectomy by Bioinformatics Analysis and *in vitro*/*vivo* Experiments

**DOI:** 10.3389/fgene.2021.670706

**Published:** 2021-06-23

**Authors:** Jian Zhao, Shi-Zhe Yu, Qiang Cai, Duo Ma, Long Jiang, Ling-Peng Yang, Zhi-Yong Yu

**Affiliations:** ^1^Department of Hepatobiliary Surgery, The Affiliated Hospital of Yunnan University, Kunming, China; ^2^Department of Surgery, The First Affiliated Hospital of Zhengzhou University, Zhengzhou, China

**Keywords:** liver regeneration, partial hepatectomy, cell cycle, UBE2C, DNA replication

## Abstract

**Background:**

The liver is the only organ that can completely regenerate after various injuries or tissue loss. There are still a large number of gene functions in liver regeneration that have not been explored. This study aimed to identify key genes in the early stage of liver regeneration in mice after partial hepatectomy (PH).

**Materials and Methods:**

We first analyzed the expression profiles of genes in mouse liver at 48 and 72 h after PH from Gene Expression Omnibus (GEO) database. Gene ontology (GO), and the Kyoto Encyclopedia of Genes and Genomes (KEGG), and protein–protein interaction (PPI) analysis were performed to identify key genes in liver regeneration. Finally, we validated key genes *in vivo* and *in vitro*.

**Results:**

We identified 46 upregulated genes and 19 downregulated genes at 48 h after PH, and 223 upregulated genes and 40 downregulated genes at 72 h after PH, respectively. These genes were mainly involved in cell cycle, DNA replication, and p53 signaling pathway. Among of these genes, cycle-related genes (Ccna2, Cdkn1a, Chek1, and Mcm5) and Ube2c were highly expressed in the residual liver both at 48 and 72 h after PH. Furthermore, Ube2c knockdown not only caused abnormal expression of Ccna2, Cdkn1a, Chek1, and Mcm5, but also inhibited transition of hepatocytes from G1 to S phase of the cell cycle *in vitro*.

**Conclusion:**

Mouse hepatocytes enter the proliferation phase at 48 h after PH. Ube2c may mediate cell proliferation by regulating or partially regulating Ccna2, Cdkn1a, Chek1, and Mcm5.

## Introduction

As an important organ for energy metabolism, bile production, and detoxification, liver is the only organ that can completely regenerate through liver cell mitosis and proliferation (Fausto et al., 2006; Song et al., 2021). This allows partial hepatectomy (PH) without affecting liver function when treating liver diseases such as primary or secondary liver tumors (Alkhalili and Berber, 2014). Studies have shown that coagulation, cytokines and growth factors secreted by inflammatory cells are the major stimulators for liver regeneration (Karin and Clevers, 2016; Isfordink et al., 2017). These factors can stimulate the transition of hepatocytes in the resting phase from G0 to G1 of the cell cycle, leading to cell proliferation (Tao et al., 2017).

Liver regeneration after PH is a complex and orderly process (Bohm et al., 2010). Mice can recover to their baseline liver quality 7–10 days after 2/3 PH, while this time for humans is 8–15 days (Michalopoulos, 2007). Understanding the regulatory mechanism of liver regeneration is of great significance to the prognosis of PH and the guiding of medication after hepatectomy. It is suggested that human umbilical cord blood mesenchymal stem cell-derived exosomal miR-124 could promote rat liver regeneration after PH via downregulating Foxg1 (Song et al., 2021). Zhang et al. (2020) pointed out that overexpression of hydroxysteroid sulfotransferase 2B1b promotes the regeneration of fatty liver after PH in mice with non-alcoholic fatty liver disease. Sirtuin 6 loss delays the cell cycle and impairs liver regeneration (Liu et al., 2019a).

A lot of studies have focused on gene regulation of liver regeneration, there are still many unexplored gene functions. Our research aimed to identify the key genes in the early stage cell proliferation of liver regeneration in mice after PH. We systematically integrated the gene expression profiles of mRNAs in the Gene Expression Omnibus (GEO) database for liver regeneration after PH to obtain differentially expressed genes. We conducted a series of analyses, including gene ontology (GO) term, and Kyoto Encyclopedia of Genes and Genomes (KEGG) pathway analysis, protein–protein interaction (PPI) analysis, to understand the potential functions of these differential genes in the process of liver regeneration. At the same time, we have verified the functions of these differential genes in the process of liver regeneration *in vivo* and *in vitro*.

## Materials and Methods

### Collection of Liver Regeneration Data From GEO

**FIGURE 1:**
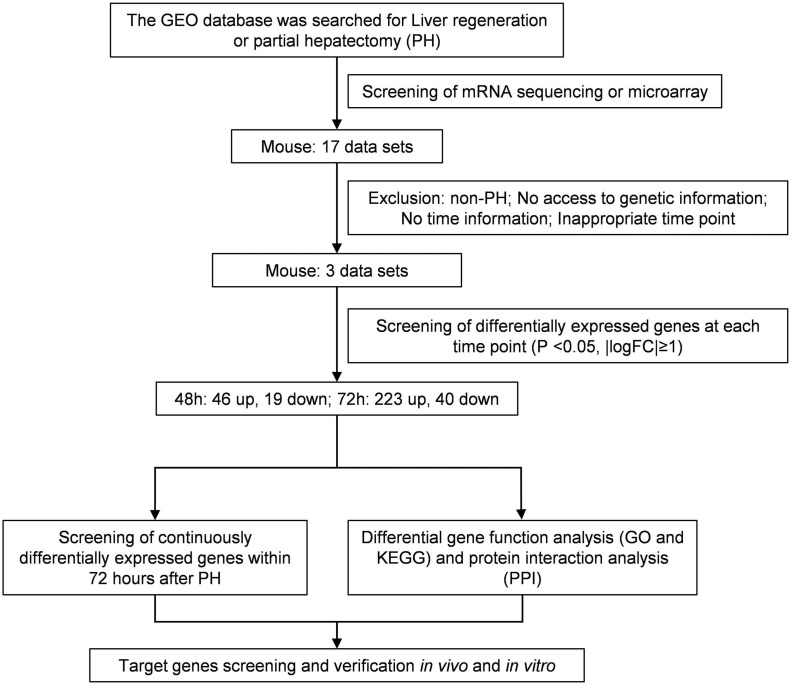
Flowchart for bioinformatics analysis of publicly available data from GEO databases.

The research in the GEO database meets the following criteria: (1) Studies of liver regeneration after PH in mice. (2) Studies of mRNAs profile. (3) Next-generation sequencing or microarray assay. (4) Liver tissues at 48 and 72 h after PH. Based on these criteria, we included three data sets of liver regeneration (GSE4528, GSE6998, and GSE20427). The microarray data of GSE4528 was based on the GPL339 ([MOE430A] Affymetrix Mouse Expression 430A Array), including two normal mouse liver tissues, two liver tissues at 48 h after PH, and two liver tissues at 72 h after PH. The microarray data of GSE6998 was based on the GPL1261 ([Mouse430_2] Affymetrix Mouse Genome 430 2.0 Array), including two normal mouse liver tissues, two liver tissues at 48 h after PH, and two liver tissues at 72 h after PH. The microarray data of GSE6998 was based on the GPL1261 ([Mouse430_2] Affymetrix Mouse Genome 430 2.0 Array), including six normal mouse liver tissues, six liver tissues at 48 h after PH. Figure 1 shows the workflow of retrieval and bioinformatics analysis of available data sets from GEO.

### Identification of Differentially Expressed Genes

The differential genes between normal liver tissues and liver tissues at 48 and 72 h after PH in each data set were analyzed using GEO2R online tool (Qian et al., 2019), *P* < 0.05 and | log fold change FC| ≥ 1 were set as the cut-off criteria. Then the consistent differential mRNAs among these three data sets were screened using Draw Venn Diagram (Dong et al., 2020)^1^.

### GO and KEGG Enrichment Analysis of Differential Genes

In order to further understand the functions of these differential genes in the process of liver regeneration, we used online software Database for Annotation, Visualization and Integrated Discovery (DAVID Bioinformatics Rescources 6.8) (Gan et al., 2018)^2^ to perform a functional enrichment analysis of the differential genes, including GO analysis [biological pathways (BP), molecular functions (MF), and cell components (CC)] and KEGG pathways analysis. Then RStudio software (version 3.5.3) (Huang et al., 2020) was used to visualize the enrichment results. The screening conditions for GO term was top 5 terms with *P* < 0.05, and the screening conditions for KEGG pathwaywas *P* < 0.05.

### PPI Network and Module Analysis

In order to study the interaction between these differential genes, we used The Search Tool for the Retrieval of Interacting Genes (STRING) (Dong et al., 2020)^3^ database to generate PPI networks. The nodes and edges in the network represented genes and their interactions, respectively. At the same time, we used Cytoscape (version 3.7.0) (Dong et al., 2020) to construct a PPI network of the target genes. The shape of the node indicated the expression level and the color indicated the degree of connection.

### Models for Liver Regeneration

Male mice aged 2–3 months were purchased from SLAC Laboratory (Shanghai, China) and kept under standard conditions (Lee et al., 2020). These mice were randomly divided into three groups: control group (*n* = 8), sham group (*n* = 8), and PH group (*n* = 8). PH surgery was mainly performed in accordance with the procedure described previously (Nevzorova et al., 2015) and the middle lobe and left lateral lobe of the mouse liver (about 2/3) were removed. The mice were euthanized at 48 h after PH, part of each liver was used to separate hepatocytes for cell cycle detection, part of each liver was fixed in 10% neutral buffered formalin for immunohistochemistry, and the rest of each liver was frozen in liquid nitrogen until use. The protocols for the care and use of animals were approved by the Animal Care and Use Committee of The Affiliated Hospital of Yunnan University.

### Cell Culture and Transfection

BNL CL.2 normal mouse liver cell line was purchased from the Cell Bank of the Chinese Academy of Sciences (Shanghai, China). The cells were cultured in high-glucose Dulbecco’s modified Eagle’s medium (Hyclone, United States) supplemented with 10% fetal bovine serum (Hyclone, United States). Cells were transfected with si-NC (100 nM) or si-Ube2c (100 nM) for 48 h using Lipofectamine 3000 (Invitrogen, United States) according to the manufacturer’s instructions. Si-NC and si-Ube2c were synthesized in GenePharma (Shanghai, China), and the sequence was shown below: Mus-si-Ube2c-1 sense: 5′-CCC ACA GCA UUU AAG AAA UTT-3′, antisense: 5′-AUU UCU UAA AUG CUG UGG GTT-3′. Mus-si-Ube2c-2 sense: 5′-AAA AAA GAC AAC ACA AAA GAG-3′, antisense: 5′-CUU UUG UGU UGU CUU UUU UCU-3′. Mus-si-Ube2c-3 sense: 5′-UAA UAU ACA UUG UUA AGG GUU-3′, antisense: 5′-CCC UUA ACA AUG UAU AUU AAA-3′. Mus-si-NC sense: 5′-UUC UCC GAA CGU GUC ACG UTT-3′, antisense: 5′-ACG UGA CAC GUU CGG AGA ATT-3′.

### Real-Time Quantitative PCR

Total RNA was extracted from cells using Trizol reagent (life, North American) and reverse-transcribed into complementary DNA using 5 × Prime Script RT Master Mix kit (Takara, Tokyo, Japan). SYBR real-time quantitative PCR (qPCR) Master Mix (Takara) was used to carry out qPCR according to the manufacturer’s instructions. Target gene expression was normalized to GAPDH levels in respective samples as an internal control and calculated using the 2^–ΔΔ*Ct*^ method. The primer sequences were shown in Table 1.

**TABLE 1:** The primers used for real-time quantitative PCR.

**Gene**	**Forward primer (5′–3′)**	**Reverse primer (5′–3′)**
Mus-Ube2c	TTACAACGCACCCACAGTGA	GTTGGGTTCTCCTAGCAGGC
Mus-Cdkn1a	AGTACTTCCTCTGCCCTGCT	GAATCTTCAGGCCGCTCAGA
Mus-Chek1	AGGAGGGAAGGCCATATCCA	CCCTATGTCTGGCTCAATTCT
Mus-Ccna2	CTGCCTTCCACTTGGCTCTC	TTGTGGCGCTTTGAGGTAGG
Mus-Bub3	CGCTTCCCTTGCCTTCAGTA	GGGCTTTGTTTCTGCGTCTG
Mus-Mcm5	GGAGGCTATTGTGCGCATTG	TGCCTCCTCTACATCAGCCT
Mus-Gapdh	AGGTCGGTGTGAACGGATTTG	TGTAGACCATGTAGTTGAGGTCA

### Western Blotting

Liver tissues or cells were lysed in RIPA buffer with complete protease inhibitor (Sigma). Nucleoprotein was prepared as previously described (Ho et al., 2010). Then whole lysates (30 μg) were separated by polyacrylamide gel electrophoresis and proteins were transferred to polyvinylidene difluoride (PVDF) membranes. These membranes were incubated with primary antibodies against BUB3 (1:2,000, CY8528), CHK1 (1:1,000, CY5063), cyclin A (1:1,000, CY1026), MCM5 (1:2,000, CY7185), P21 (1:1,000, 27296-1-AP), Ube2c (1:1,000, 12134-2-AP), and GAPDH (1:2,000, ab9485) overnight at 4°C followed by incubate with secondary antibodies [goat anti-rabbit secondary antibody (HRP)] 2 h at room temperature. Immune complexes were detected using the Enhanced Chemiluminescence System (Thermo Fisher Scientific) and the intensity of each band was quantified using ImageJ software. Primary antibodies against Bub3, CHK1, cyclin A, and MCM5 were purchased from Abways (Shanghai, China). Primary antibodies against P21 and Ube2c were purchased from Proteintech (Wuhan, Hubei, China). Primary antibody against GAPDH was purchased from Abcam (Shanghai, China).

### Immunohistochemical Staining

The 10% neutral buffered formalin fixed and paraffin-embedded liver tissues were used to prepare liver sections (4 μm). Sections were pre-treated using heat mediated antigen retrieval with sodium citrate buffer (pH 6) for 20 min. Then these sections were incubated with primary antibody overnight at 4°C and secondary antibody for 2 h at room temperature. The primary antibodies included BUB3 (1:100), CHK1 (1:50), cyclin A (1:50), MCM5 (1:100), P21 (1:100), Ube2c (1:200), and PCNA (1:5,000, 13110S, CST). Positive hepatocytes were observed and captured at 200× under microscopy.

### CCK-8 Assay

BNL CL.2 normal mouse liver cells (2 × 10^3^ cells/well) were treated with si-Ube2c for 72 h in 96-well plates. According to the manufacturer’s protocol, Cell Counting Kit-8 (C0037, Beyotime, Shanghai, China) was used to detect cell viability at 0, 24, 48, and 72 h after treatment. A microplate reader (Multiskan^TM^ FC, Thermo Fisher Scientific) was used to measure the absorbance at 450 nm.

### Flow Cytometry

Cell Cycle and Apoptosis Analysis Kit (C1052) (Beyotime, Shanghai, China) was used to perform the flow cytometry and assess cell cycle progression according to the manufacturer’s protocol. In general, the cells were fixed in an ice bath pre-cooled 70% ethanol for 2 h. Then 0.5 mL of propidium iodide staining solution was added to each tube of cell samples, and keep in the dark at 37°C for 30 min. A flow cytometer (BD FACSCalibur) was used to detect the red fluorescence at the excitation wavelength of 488 nm. FlowJo V10 software was used for cell DNA content analysis.

### Statistical Analysis

Data were shown as mean ± standard deviation (SD). Statistical analysis was performed using one-way analysis of variance (ANOVA) by GraphPad Prism 8.0 statistical software (GraphPad Software, CA, United States) and significant differences were defined when *P* < 0.05.

## Results

### Convergence of Differential Genes at 48 h After PH and Function Analysis Across Different Studies From GEO Database

**FIGURE 2:**
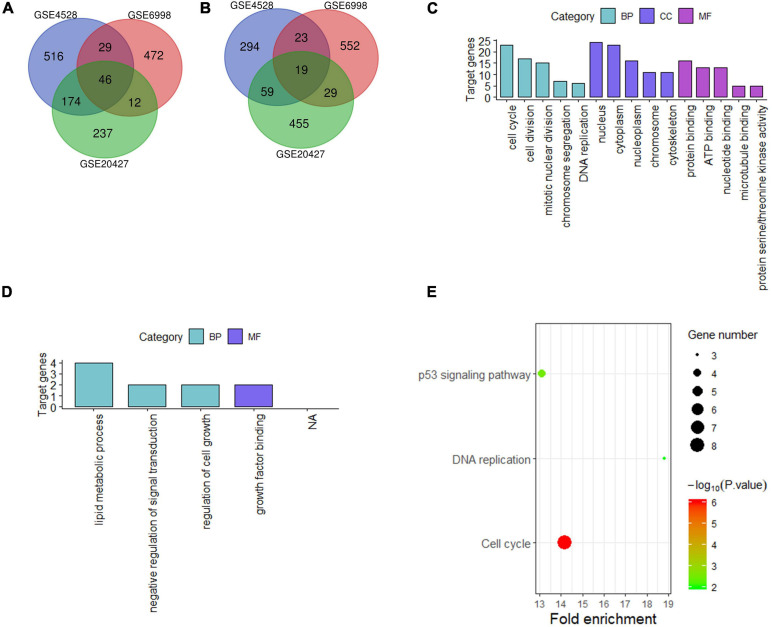
Analysis of differentially expressed genes in mouse Liver at 48 h after PH. **(A,B)** Venn diagram of differentially expressed genes (up and down). **(C,D)** GO analysis (top five terms) (up and down); **(E)** KEGG analysis of all differential genes (*P* < 0.05).

For liver tissue at 48 h after PH, 1,160 differential genes in GSE4528, 1,182 differential genes in GSE6998, and 1,031 differential genes in GSE20427 were identified. Among the differential genes, 765, 559, and 469 genes were upregulated (Figure 2A) while 395, 623, and 562 genes were downregulated in GSE4528, GSE6998, and GSE20427, respectively (Figure 2B). The consistently upregulated and downregulated genes in all three independent cohorts were identified using Venn analysis and a Venn diagram was generated by Draw Venn Diagram. As a result, we got a total of 46 upregulated genes and 19 downregulated genes.

These 65 differential genes were submitted into DAVID for GO analysis and KEGG pathway analysis to gain insights into the biological functions of these consensus genes in residual liver. In BP term, the result demonstrated that these upregulated genes were mainly enriched in cell cycle, cell division, mitotic nuclear division; In CC term, these upregulated genes were mainly involved in nucleus, cytoplasm, nucleoplasm; In MF term, these upregulated genes were mainly associated with protein binding, ATP binding and nucleotide binding (Figure 2C). Downregulated genes were mainly enriched in lipid metabolic process, negative regulation of signal transduction and regulation of cell growth in BP term, and mainly associated with growth factor binding in MF term (Figure 2D). KEGG pathway analysis indicated that these 65 differential genes were mainly related to cell cycle, DNA replication and p53 signaling pathway (Figure 2E).

### Convergence of Differential Genes at 72 h After PH and Function Analysis Across Different Studies From GEO Database

**FIGURE 3:**
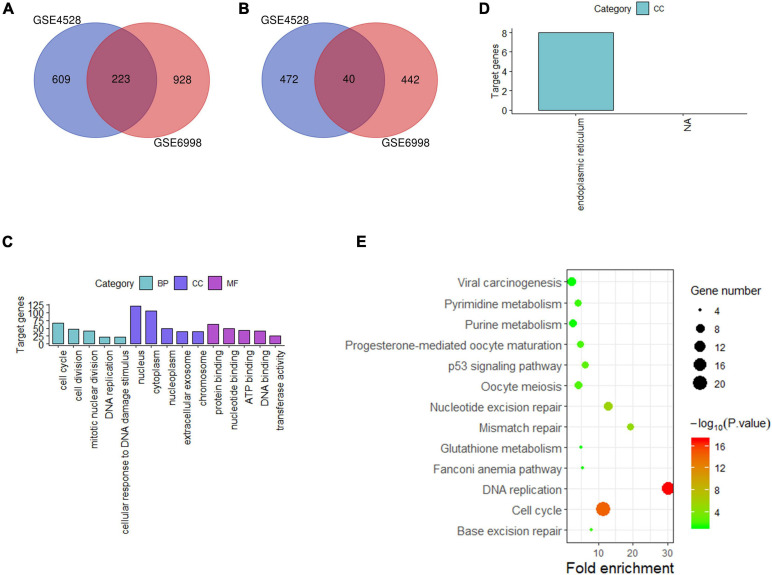
Analysis of differentially expressed genes in mouse Liver at 72 h after PH. **(A,B)** Venn diagram of differentially expressed genes (up and down). **(C,D)** GO analysis (top five terms) (up and down); **(E)** KEGG analysis of all differential genes (*P* < 0.05).

For liver tissue at 72 h after PH, 1,344 differential genes in GSE4528 and 1,633 differential genes in GSE6998 were identified. Among the differential genes, 832 and 1,151 genes were upregulated (Figure 3A) while 512 and 482 genes were downregulated in GSE4528 and GSE6998, respectively (Figure 3B). Venn analysis showed a total of 223 upregulated genes and 40 downregulated genes.

These 263 differential genes were also submitted into DAVID for GO analysis and KEGG pathway analysis. In BP term, the result demonstrated that these upregulated genes were mainly enriched in cell cycle, cell division, and mitotic nuclear division; In CC term, these upregulated genes were mainly involved in nucleus, cytoplasm, nucleoplasm; In MF term, these upregulated genes were mainly associated with protein binding, nucleotide binding and ATP binding (Figure 3C). Downregulated genes were mainly involved in endoplasmic reticulum in CC term (Figure 3D). KEGG pathway analysis indicated that these 263 differential genes were mainly related to cell cycle, DNA replication and nucleotide excision repair (Figure 3E).

### Network Analyses of These Consensus Genes

**FIGURE 4:**
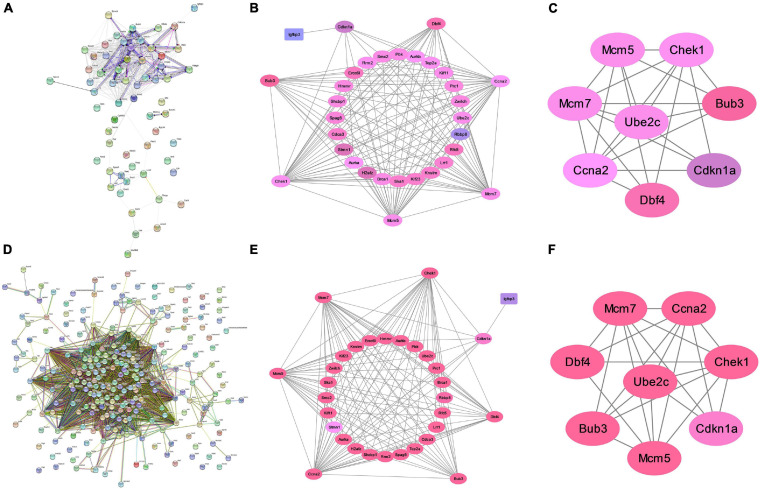
Analysis of protein interaction of differential genes after PH in mice. **(A)** Protein–protein interaction network of differential genes at 48 h after PH. **(B)** Genes directly linked to cell cycle related genes at 48 h after PH. **(C)** Ube2c were interacted with cell cycle genes other than Dbf4 at 48 h after PH. **(D)** Protein–protein interaction network of differential genes at 72 h after PH. **(E)** Genes directly linked to cell cycle related genes at 72 h after PH. **(F)** Ube2c were interacted with cell cycle genes other than Dbf4 at 72 h after PH.

We constructed a PPI network to further explore the interaction between the consensus genes by using STRING database and Cytoscape. As shown in Figure 4A, there were 61 nodes and 397 edges in the network between consensus genes in residual liver at 48 h after PH. The top 10 hub genes were: Ccna2, Aurkb, Aurka, Top2a, Kif11, Smc2, Pbk, Zwilch, Mcm5, and Rrm2. There were 243 nodes and 4,456 edges in the network between consensus genes in residual liver at 72 h after PH. The top 10 hub genes were: Zwilch, Spc25, Pbk, Trip13, Uhrf1, Kif11, Aurka, Aurkb, Rrm2, and Plk1 (Figure 4D). We analyzed the differential genes involved in cell cycle pathway, which was the most active pathway at 48 and 72 h after PH. The results showed that a total of 8 upregulated genes and 20 upregulated genes in cell cycle at 48 and 72 h after PH, respectively (Table 2). Venn analysis showed a total of 44 upregulated consensus genes and 8 downregulated consensus genes at 48 and 72 h after PH (Table 3), and Ccna2, Cdkn1a, Pbl1, Dbf4, Mcm7, Chek1, Mcm5, and Bub3 were both involved in cell cycle. Then PPI network showed 25 consensus genes were related to 6 genes of the cell cycle both at 48 and 72 h after PH. Among these 25 genes, only Igfbp3 was downregulated (Figures 4B,E). Furthermore, we found that only Ube2c were interacted with the six genes other than *DBF4* (Figures 4C,F).

**TABLE 2:** The differently expressed genes involved in cell cycle pathway in mouse liver at 48 and 72 h after PH.

**Pathway name**	**Time**	**Gene names**	
		**Up**	**Down**
Cell cycle	48 h	Ccna2, Cdkn1a, Rbl1, Dbf4, Mcm7, Chek1, Mcm5, Bub3	-
	72 h	Cdkn1a, Pcna, Mcm7, Plk1, Ttk, Cdc25c, Ccna2, Cdc20, Dbf4, Ccne2, Rad21, Chek1, Cdk1, Mcm3, Mcm4, Mcm5, Mcm6, Bub3, Mcm2, Mad2l1	-

**TABLE 3:** Genes that are abnormally expressed in mouse liver at 48 and 72 h after PH.

**Regulation**	**Gene name**
Up	Smc2, Cdca3, Rfc5, Rbbp8, Orm2, Chek1, Ube2c, Shcbp1, Aurka, Stmn1, Gm5593///Ccnb1, Ccna2, Lcn2, Saa2, Ercc6l, Prc1, Spag5, Hmmr, Mcm5, Kif11, Aurkb, Zwilch, Cdkn1a, Cyb561, Mtnr1a, Ier5, Kif23, Pbk, Thbd, Dbf4, Top2a, Mcm7, Saa1, Bub3, Gja1, Ska1, Brca1, H2afz, Apoa4, Mt2, Lrr1, Prtn3, Knstrn, Rrm2
Down	Hsd3b5, Pklr, Igfbp3, Socs2, Car3, Ces1e, Aacs, Nrep

### Active DNA Synthesis in Residual Liver at 48 h After PH

**FIGURE 5:**
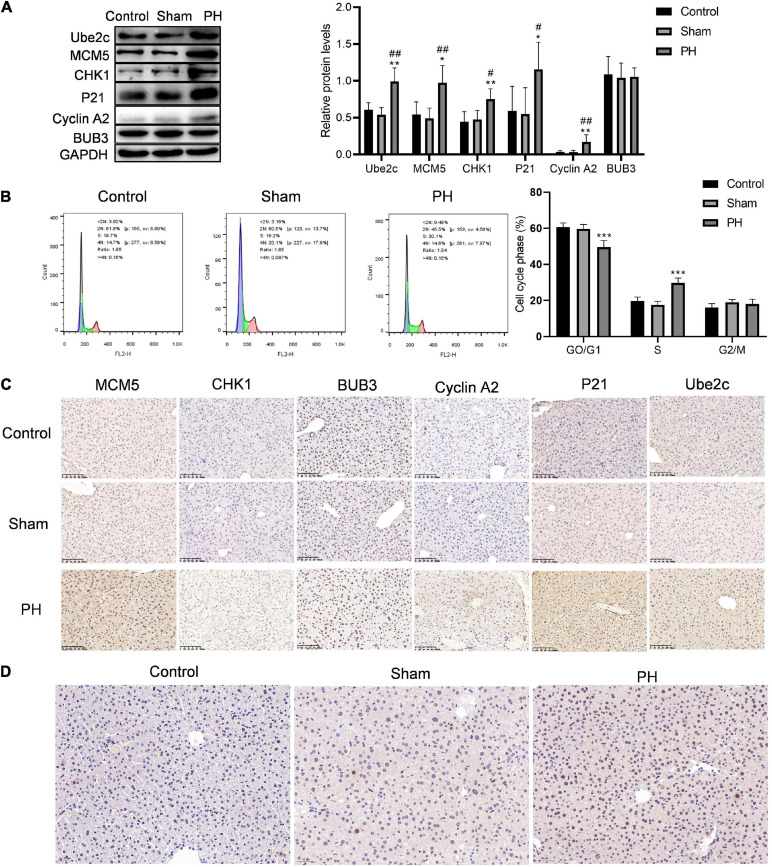
Hepatocytes convert from G1 phase to S phase of the cell cycle when faced with PH. **(A)** Target genes in cell cycle were verified using western blotting *in vitro*; ***P < 0.001 compared to control group; ###*P* < 0.001 compared to sham group. **(B)** Cell cycle of hepatocytes after PH were detected using flow cytometry; ***P < 0.001 compared to other groups. **(C)** Immunohistochemical staining of target proteins in mice livers at 48 h after PH (original magnification: ×200, scale bars represent 100 μm). **(D)** Representative liver sections stained with PCNA in mice at 48 h after PH (original magnification: ×200, scale bars represent 50 μm).

We constructed a mouse model for liver regeneration to verify the expression of target genes in residual liver at 48 h after PH. Western blotting results showed that protein levels of Ube2c, Cyclin A2, P21, CHK1, and MCM5 were both significantly increased in the residual liver at 48 h after PH (Figure 5A). Immunohistochemical staining results were similar to the results of western blotting (Figure 5B). At the same time, we used flow cytometry to detect the cell cycle of hepatocyte during liver regeneration. The results showed that the proportion of G0/G1 cells in PH group was obviously decreased compared to that in control group and sham group, while the proportion of S cells in PH group was obviously increased compared to that in control group and sham group (Figure 5C). In addition, the level of PCNA in PH group was also higher than that in control and sham group (Figure 5D). This indicates that PH stimulates the remaining liver cells to synthesize DNA, enter the cell cycle, and promote hepatocyte proliferation at 48 h.

### Decreased Ube2c Delays Hepatocyte Transition Into S Phase at 48 h After PH

**FIGURE 6:**
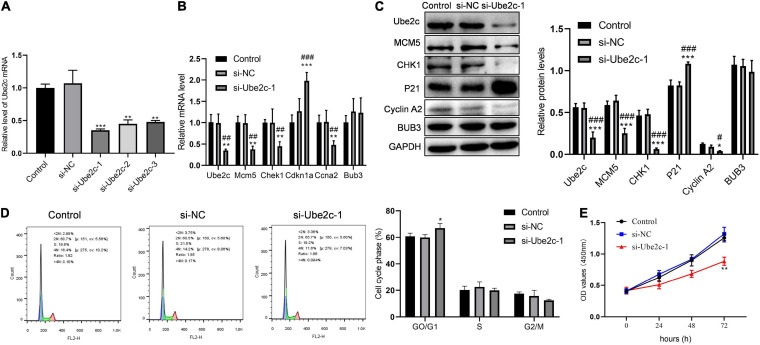
Inhibitory effect of knockdown Ube2c on hepatocyte proliferation *in vitro.* **(A)** The gene expression level of Ube2c knockdown on hepatocytes after transfection of siRNA; **P < 0.01 compared to other groups. **(B,C)** Transcription and protein levels of target genes in Ube2c knockdown hepatocytes; *P < 0.05, **P < 0.01, ***P < 0.001 compared to control group; #*P* < 0.05, ##*P* < 0.01, ###*P* < 0.001 compared to si-NC group. **(D)** Cell cycle of hepatocytes with Ube2c knockdown; *P < 0.05 compared to other groups. **(E)** CCK8 assay was performed to detect the proliferation of hepatocytes containing si-Ube2c.

We used qPCR to detect the interference of Ube2c and the effect of knocking down Ube2c on the expression of cycle-related genes. Compared with the control group and the si-NC group, si-Ube2c-1 has the best interference effect on Ube2c mRNA (Figure 6A). The decreased Ube2c mRNA caused a significant reduction in the transcription level of Mcm5, Chek1, and Ccna2, while the transcription level of Cdkn1a increased significantly (Figure 6B). The western blotting results also confirmed that the decrease of Ube2c protein was accompanied by the decrease of MCM5, CHK1, cyclin A2 proteins and the increase of P21 protein (Figure 6C). The results of flow cytometry showed that Ube2c knockdown increased the proportion of GO/G1 cells during liver regeneration (Figure 6D). Compared with the control group and the si-NC group, the cell viability of the si-Ube2c treatment group also decreased with time (Figure 6E). This indicates that Ube2c knockdown has an inhibitory effect on hepatocyte proliferation during liver regeneration.

## Discussion

Liver regeneration occurs after surgery, trauma, infection, liver transplantation and other events that may lead to the loss of liver mass (Li et al., 2017). Liver regeneration helps the liver to recover from the symptoms of injury, and poor liver regeneration may lead to fatality (Dutkowski et al., 2015). Clarifying the functional mechanism of liver regeneration is of great significance for improving the clinical potential of postoperative liver regeneration. In this study, 65 differential genes (46 up-regulated and 19 down-regulated) and 263 differential genes (223 up-regulated and 40 down-regulated) were identified, respectively, at 48 and 72 h after PH from three gene expression profile data sets. Ube2c and six cell-cycle related genes were integrated through function analysis and PPI network. The function analysis of these target genes was performed *in vivo* and *in vitro*.

There are two modes of liver regeneration caused by liver tissue loss. One is that after PH, quiescent hepatocytes enter into the cell cycle and proliferate to compensate for the loss of liver tissue (Sun and Irvine, 2014); the other is induced by toxin or viral infection, in this case, hepatocytes are damaged and liver regeneration is based on the differentiation of oval cells into hepatocytes and biliary cells (Tao et al., 2017). Our research showed that at 48 h after PH, 46 genes were up-regulated, and 8 of them were enriched in the cell cycle signaling pathway. At 72 h after PH, there were 223 genes up-regulated, and 20 of them were enriched in the cell cycle signaling pathway. At the same time, the results of animal models showed that the ratio of G0/G1 cells was down-regulated at 48 h after PH, while the ratio of S-phase cells was up-regulated. These results suggest that hepatocytes enter a proliferation state in large numbers at 48 h after PH. In addition, Ccna2, Cdkn1a, Dbf4, Mcm7, Chek1, Mcm5, and Bub3 showed a significant increase at 48 and 72 h after PH. Our results of animal model verified that Ccna2, Cdkn1a, Chek1, and Mcm5 were significantly up-regulated at 48 h after PH. While Ccna2 (Li et al., 2019), Chek1 (Goto et al., 2019), and Mcm5 (Simonetti et al., 2019) play an important role in promoting the cell cycle process. It is speculated that Ccna2, Chek1, and Mcm5 are important factors that promote liver regeneration at 48 h after PH.

Ube2c is considered to be a gene related to chromosomal instability and is overexpressed in solid tumors (Carter et al., 2006). Ube2c showed a significantly up-regulated abnormal expression at 48 and 72 h after PH in this study, and there is a connection with cycle genes Ccna2, Chek1, Cdkn1a, and Mcm5, indicating that Ube2c is also involved in cell proliferation during liver regeneration (Maillet et al., 2018). After interfering with Ube2c, the hepatocyte cycle was blocked, which verified the positive role of Ube2c in the proliferation of hepatocytes. In addition, the decrease in Ube2c caused a decrease in Ccna2, Chek1, and Mcm5 and an increase in Cdkn1a in hepatocytes. The initiation of Cdkn1a expression, cyclin-dependent kinase inhibitor 1A, leads to cell G1/S arrest (Kleinsimon et al., 2018). Ube2c may promote hepatocytes to enter the cell cycle by up-regulating cell cyclins (Cyclin A, Chek1, and MCM5) and down-regulating the cycle kinase inhibitor P21.

In addition, we also found that with the prolongation of PH postoperative time (from 48 to 72 h), Ube2c was closely associated with genes related to the p53 signaling, DNA replication, and DNA repair pathways. Xie et al. found that UBE2C is related to the cell cycle, cell proliferation and division of mouse lung mesenchymal progenitor cells (Xie et al., 2018). Maillet et al. (2018) found that UBE2C is involved in the mid-term regulation of hepatocyte cell cycle. Some cancer studies have shown that UBE2C is highly expressed in cancer cells and is involved in cell proliferation, mitosis, and G2/M transformation in cell cycle together with STMN1, AURKA, TOP2A, KIF11, PLK1, and CDK1 (Campone et al., 2008; Gu et al., 2017; Liu et al., 2019b; Li et al., 2020). This relationship between these genes and Ube2c was also found in our research, and we also detected that PCNA is highly expressed in the liver tissue of mice with PH. This indicates that Ube2c may have a multi-directional promotion effect on hepatocyte proliferation. Studies on the mechanism of UBE2C expression indicate that the expression of UBE2C may be stimulated by androgen- or estrogen-receptor, lncRNA and miRNA (Kato et al., 2016; Gu et al., 2017; Hu et al., 2019; Liu et al., 2020). These provide a general direction for us to further study the reasons for the up-regulation of Ube2c in the early stage of liver regeneration.

In summary, our study showed the high-level cell cycle related genes (Ccna2, Cdkn1a, Mcm7, Chek1, Mcm5, and Bub3) were associated with Ube2c both at 48 and 72 h after PH. The effect of Ube2c on hepatocyte proliferation *in vivo* and *in vitro* may be related to MCM5, Chek1, Cyclin A2, and P21.

## Data Availability Statement

The datasets presented in this study can be found in online repositories. The names of the repository/repositories and accession number(s) can be found in the article/Supplementary Material.

## Ethics Statement

The animal study was reviewed and approved by The Affiliated Hospital of Yunnan University.

## Author Contributions

Z-YY: conceptualization, design, project administration, and supervision. JZ, S-ZY, and QC: data curation, formal analysis, investigation, methodology, and writing – original draft. DM, LJ, and L-PY: resources, software, validation, visualization, and writing – review and editing. All authors read and approved the final version of the manuscript.

## Conflict of Interest

The authors declare that the research was conducted in the absence of any commercial or financial relationships that could be construed as a potential conflict of interest.

## Footnotes

1 http://bioinformatics.psb.ugent.be/webtools/Venn/

2 https://david.ncifcrf.gov/

3 https://string-db.org/
