# Supplementary figures and images for: Identifying the Key Genes in Mouse Liver Regeneration After Partial Hepatectomy by Bioinformatics Analysis and in vitro/vivo Experiments
Source: Front Genet. 2021 Jun 23;12:670706. doi: 10.3389/fgene.2021.670706 (PMC8260846; doi:10.3389/fgene.2021.670706)

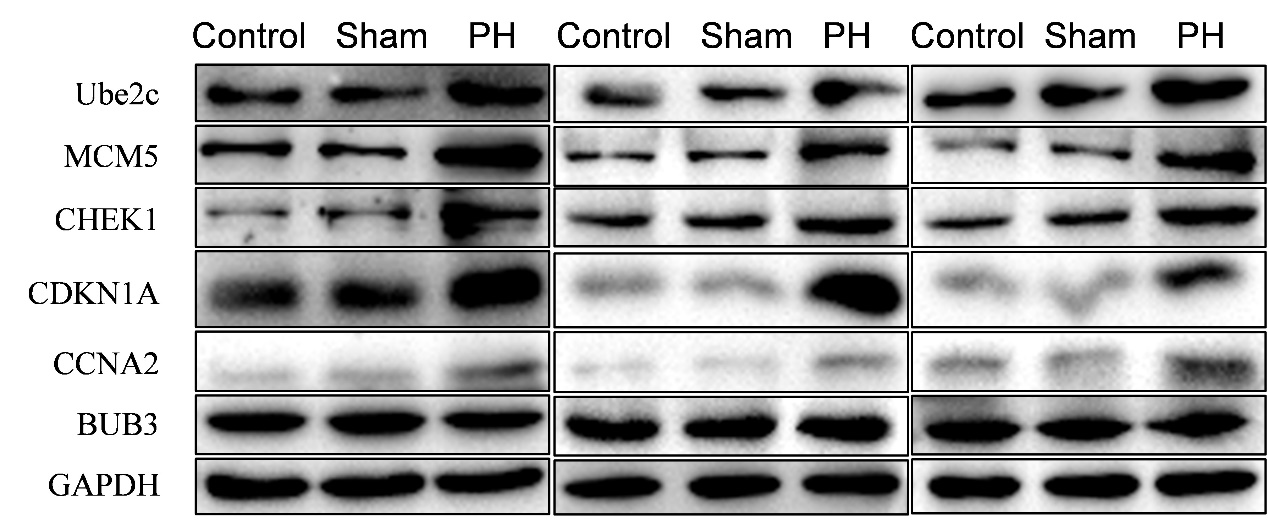

Supplement: Supplementary file 1 [file Image_1.JPEG]
